# Time for a reassessment of the treatment of hypothyroidism

**DOI:** 10.1186/s12902-019-0365-4

**Published:** 2019-04-18

**Authors:** John E. M. Midgley, Anthony D. Toft, Rolf Larisch, Johannes W. Dietrich, Rudolf Hoermann

**Affiliations:** 1 North Lakes Clinical, 20 Wheatley Avenue, Ilkley, LS29 8PT UK; 2 Spire Murrayfield Hospital, Edinburgh, EH12 6UD UK; 3 Department for Nuclear Medicine, Klinikum Lüdenscheid, Paulmannshöherstr. 14, 58515 Lüdenscheid, Germany; 4 Medical Department I, Endocrinology and Diabetology, Bergmannsheil University Hospitals, Ruhr University of Bochum, Buerkle-de-la-Camp-Platz 1, D-44789 Bochum, Germany; 5 Ruhr Center for Rare Diseases (CeSER), Ruhr University of Bochum and Witten/Herdecke University, Alexandrinenstr. 5, D-44791 Bochum, Germany; 6 113 Andersons Road, Yandina, QLD 4561 Australia

**Keywords:** Thyroxine therapy, Free thyroxine, Free triiodothyronine, Thyroid stimulating hormone, Diagnostic strategies, Treatment protocols, Randomised clinical trials, Statistical analysis

## Abstract

**Background:**

In the treatment for hypothyroidism, a historically symptom-orientated approach has given way to reliance on a single biochemical parameter, thyroid stimulating hormone (TSH).

**Main body:**

The historical developments and motivation leading to that decision and its potential implications are explored from pathophysiological, clinical and statistical viewpoints. An increasing frequency of hypothyroid-like complaints is noted in patients in the wake of this directional shift, together with relaxation of treatment targets. Recent prospective and retrospective studies suggested a changing pattern in patient complaints associated with recent guideline-led low-dose policies. A resulting dramatic rise has ensued in patients, expressing in various ways dissatisfaction with the standard treatment. Contributing factors may include raised problem awareness, overlap of thyroid-related complaints with numerous non-specific symptoms, and apparent deficiencies in the diagnostic process itself. Assuming that maintaining TSH anywhere within its broad reference limits may achieve a satisfactory outcome is challenged. The interrelationship between TSH, free thyroxine (FT4) and free triiodothyronine (FT3) is patient specific and highly individual. Population-based statistical analysis is therefore subject to amalgamation problems (Simpson’s paradox, collider stratification bias). This invalidates group-averaged and range-bound approaches, rather demanding a subject-related statistical approach. Randomised clinical trial (RCT) outcomes may be equally distorted by intra-class clustering. Analytical distinction between an averaged versus typical outcome becomes clinically relevant, because doctors and patients are more interested in the latter. It follows that population-based diagnostic cut-offs for TSH may not be an appropriate treatment target. Studies relating TSH and thyroid hormone concentrations to adverse effects such as osteoporosis and atrial fibrillation invite similar caveats, as measuring TSH within the euthyroid range cannot substitute for FT4 and FT3 concentrations in the risk assessment. Direct markers of thyroid tissue effects and thyroid-specific quality of life instruments are required, but need methodological improvement.

**Conclusion:**

It appears that we are witnessing a consequential historic shift in the treatment of thyroid disease, driven by over-reliance on a single laboratory parameter TSH. The focus on biochemistry rather than patient symptom relief should be re-assessed. A joint consideration together with a more personalized approach may be required to address the recent surge in patient complaint rates.

## Background

The clinical state of hypothyroidism (then known as myxoedema) was described around 1870, and 10 years later it was recognised as being due to loss of function of the thyroid gland [1–4]. While the Chinese may have been treating goitre in cretins with sheep’s thyroid in the sixth century BCE [5], initial attempts at treating hypothyroidism were made by transplantation of animal thyroid tissue, followed by injectable and oral formulations [5–7]. In 1914, Kendall [8] was the first to purify the hormone thyroxine at Mayo Laboratories, which was synthesised as levothyroxine (LT4) in 1926 [9]. Despite this early chemical breakthrough in drug manufacturing, desiccated animal thyroid extract remained widely used, and even at this time some patients still regard it as the most satisfactory treatment of hypothyroidism for them [10, 11]. A policy was adopted by endocrinologists in the 1960s to replace thyroid extract with synthetic levothyroxine as the latter was then more consistent in its content [12–15]. More recently, thyroid extracts have been standardized by modern high pressure liquid chromatography (HPLC) techniques to maintain their content of thyroid hormones in different batches within USP specifications. Few clinical trials have been performed to compare the efficacy of the two products [15], and an exploratory RCT was conducted only in 2013 [16]. From its very beginnings replacement therapy was individualised and guided by the measurement of basal metabolic rate, a peripheral marker of the adequacy of thyroid hormone action [17]. Such a test was cumbersome and operator-dependent and was supplanted by biochemical tests such as protein-bound iodine measurements initially in the 1950s, followed in the early 1970s by radioimmunoassay methods for measuring serum concentrations of T3, T4 and TSH [18–21].

Despite a historically late start in its recognition as a disease entity, hypothyroidism has remarkably become one of the most frequently diagnosed diseases in the Western world, and levothyroxine one of the most frequently used drugs worldwide [22–24].

How could a condition that had been overlooked throughout the centuries of human culture rise to such prominence in such a short time period? Clearly, this was related to the convenient and sensitive measurement of serum TSH [25], which has achieved a pre-eminent position in defining primary hypothyroidism [26]. Consequently, a new disease class of subclinical hypothyroidism was introduced, which is solely based on the presence of an elevated TSH while the thyroid hormones FT3 and FT4 remain within their respective reference ranges [26]. This strategy has not remained unchallenged and the deficiencies of this diagnostic approach have been reviewed elsewhere [27]. In an attempt to scale back on the avalanche of purported thyroid diseases created by this strategy, the TSH threshold for treatment was raised in recent guidelines [26]. In doing so, another problem was created by dissociating the TSH-based diagnosis of the disease from the requirement of therapeutic intervention. However, doctors and patients find it incomprehensible that a thyroid condition labelled as a disease would not therefore require suitable intervention. This questions the practical value of designation and appropriateness of the current diagnostic entity of subclinical hypothyroidism.

## Main text

In this article, we take a closer look how these technical changes may have impacted on patient care. A transition occurred from the era of low metabolic rate regarded as synonymous with hypothyroidism to a purely biochemically based definition [28]. Hence, TSH measurement became the new determinant of hypothyroidism [26]. Consequently, treatment habits changed over the last decade and were more related to laboratory records than subjective patient experience. In particular, LT4 replacement doses tended to decrease, as a suppressed TSH was viewed as evidence of overtreatment [24, 26]. However, for two reasons this is an area of considerable uncertainty. Firstly, thyroid-related patient complaints overlap with a plethora of non-specific symptoms caused by other conditions and diseases [29–36]. Thyroid tests are also more likely to be obtained in patients with unspecific symptoms [37–39]. In these conditions, LT4 treatment may not be superior to placebo in symptom alleviation [40–42]. Secondly, TSH is increasingly recognised to be less reliable as a definitive diagnostic tool than previously assumed [27]. Not only is its reference interval not universally agreed on or adjusted for various influences, such as ethnicity, iodine supply, age, but the univariate statistical derivation of a TSH reference range is inherently ill-defined owing to its nature as a controlling element [43]. Physiologically, stimulation by TSH raises thyroid hormones to a level appropriate to the optimal well-being of a person. Because TSH, FT4 and FT3 are interrelated through the operation of hypothalamic-pituitary-thyroid feedback regulation, integrated pairs of TSH and FT4 values define the so-called individual set points [43, 44]. Unlike a population-based univariate reference interval, set points are subject to multivariate normality and narrow homeostatic ranges [43]. When plotting TSH against FT4 concentrations the resulting distribution in a healthy population does not describe the familiar rectangle, but a kite-shaped area [43]. Accordingly, a TSH value can be indicative of true euthyroidism in an individual despite it slightly exceeding the upper reference limit, while a TSH measurement within that reference interval may represent a truly hypothyroid subject [43]. Isolated TSH interpretation thereby becomes ambiguous, resulting in unacceptable diagnostic and therapeutic uncertainty surrounding a given TSH measurement when it approaches the TSH euthyroid range [43, 44]. As a consequence, this strategy divorces diagnostic disease definitions from treatment targets. Rationally therefore, the triple roles of TSH as a screening test, diagnostic tool and therapeutic target require separate assessment. Diagnostic reliability for patients may be improved by reconstructing personal TSH-FT4 set points, depending on whether this novel approach can be confirmed in clinical trials [45].

**Fig. 1:**
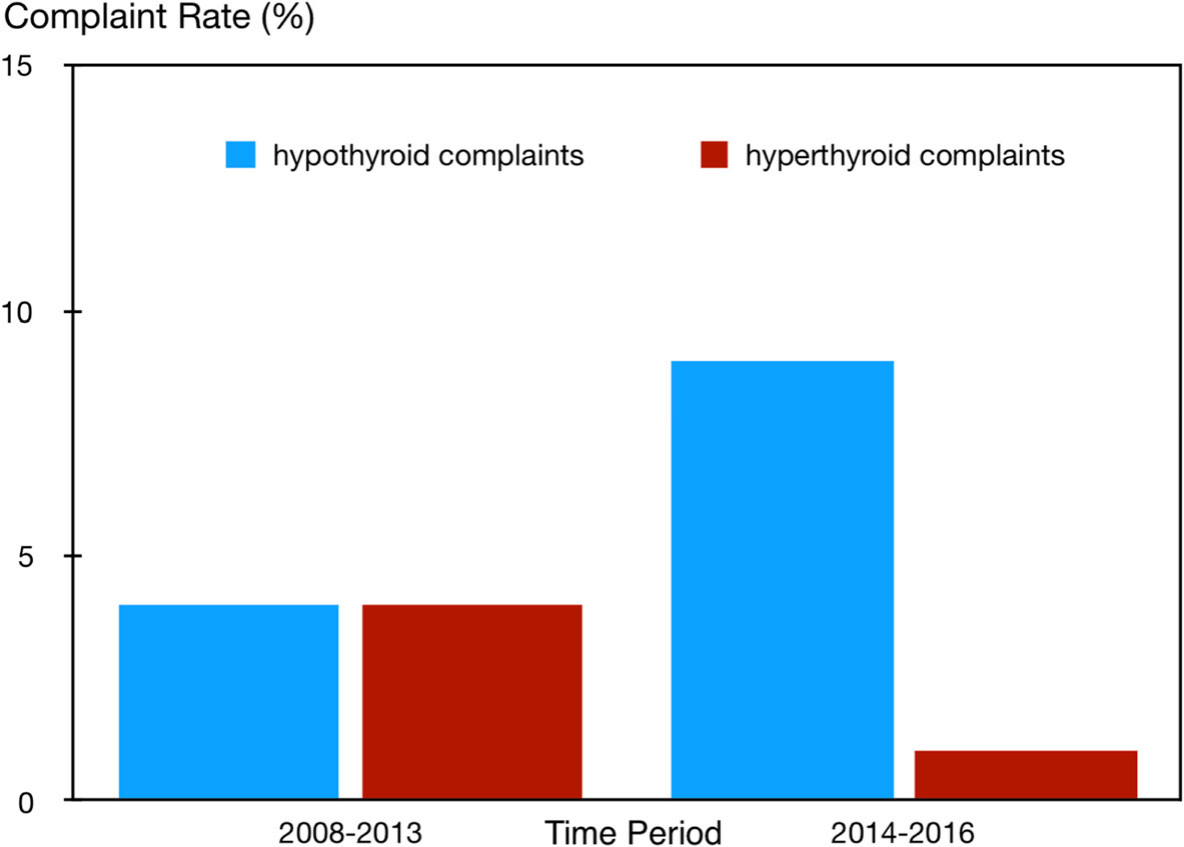
Relative rates of hypothyroid versus hyperthyroid complaints reported by patients followed on LT4-treatment for differentiated thyroid carcinoma in two time periods. Trends in hypothyroid and hyperthyroid complaints have reversed over the last decade, in association with relaxation of TSH-suppression, marking an important change in the treatment strategy (see text). Data are from a retrospective study involving 2309 visits of 319 patients [50]

Both the non-specific nature of complaints and inherent deficiencies in the diagnostic process raise an unsettling dilemma for patients and thyroid specialists alike. The issues are exemplified and particularly pertinent to an etiological disease entity whose consequences are paralleled in similar outcomes: primary hypothyroidism due to total thyroidectomy in patients with differentiated thyroid cancer. Treatment requirements and dosing of the drug LT4 changed when guidelines relaxed the need for TSH-suppressive treatment targets for these patients [46, 47]. The reason for this shift was not primarily motivated by any improvement in the replacement strategy but by a revision of the long-held tenet that TSH may act as a thyroid growth stimulating hormone. Even when only present at a low level in the circulation it was believed that it could potentially stimulate the growth of remaining tumour cells and thereby promote the relapse of the thyroid cancer in the long-term [48]. This view has recently been revised, and TSH suppression is now deemed unnecessary for many thyroid cancer patients [49]. However, this remarkable strategy change has presented a unique opportunity to study the implications for patient complaints of such a far-reaching decision to abandon TSH-suppressive LT4 treatment in low-risk thyroid cancer [47]. Although this could not be done in a prospective study, careful retrospective analysis revealed some interesting trends [50]. Over the years when replacement therapy aimed at complete TSH suppression a relatively low rate of persistent hypothyroid complaints was reported by patients followed at a single institution, much lower than in the subsequent years when the relaxed TSH policy came into effect (Fig. 1). The reverse was true for hyperthyroid complaints reported by patients, which were relatively higher in the first and much lower in the second time period (Fig. 1). The symptom reporting by these patients indicates a historical shift in the trend from a lack of hypothyroid symptoms on LT4 towards an increased awareness of the persisting symptomatology. While the nature, reliability and accuracy of freely communicated symptoms may be questionable it appears that the opposing trends in these rates in the same patients are well documented in this cohort, and they occurred in association with an important change in the treatment policy during follow-up [46–50]. We are not aware of any prospective studies that followed this historic shift in the pattern of patient complaints during the last decade. The changing pattern in patient complaints observed in this cohort [50] and associated with the low-dose policy promoted by recent guidelines is mirrored in a recent prospective study [51] and by a dramatic increase in patients worldwide expressing their concerns and dissatisfaction with the standard treatment in various ways including over the internet and through patient advocacies [52]. This sentiment was confirmed by a large online survey of 12,146 hypothyroid patients conducted by the American Thyroid Association [11].

The expression of dissatisfaction may be partly explained by raised awareness of the problem, based on unspecific subjective criteria, and the possible contribution of a lack in certainty of the diagnostic process discussed above [11, 29–45]. Patient expectations introduce a confounding influence on perceived outcomes [11, 53, 54]. This is difficult to address, particularly since expectation bias extends to RCTs, regarded as the highest class of evidence in Evidenced-Based Medicine [53]. A conflict arises between Evidenced-Based Medicine and FDA regulations, the latter mandating that drug evaluation is strictly done under conditions of actual use [53, 55]. A statistical remedy (R2R) has been proposed to adjust for expectation bias, but we are not aware of any thyroid-related analysis following such a rigorous protocol [53].

A question may be asked as to why such a renunciation of a previous protocol has not been accompanied by the initiation of appropriate trials to monitor the consequences of the new recommendations and the transition period in a suitable way. We strongly believe that this should become a priority from a public health perspective and an important joint task of the stakeholders advocating for change in the best interest of patients. This would make any discussion surrounding this important topic better grounded in evidence. As most of our patients were otherwise healthy and free of comorbidity it does not seem to be plausible or fair to blame a host of other possible influences for their complaints [50]. Similarly, in patients with autoimmune thyroiditis, LT4 treatment did not restore quality of life assessed with a validated state-of-the-art and thyroid-specific instrument to that of the healthy population in a large Danish open label study [51]. It remains however questionable whether these patients received optimum treatment, since some patients did not have their TSH “normalised” and the pituitary hormone may also be an unreliable marker in this particular setting [51].

**Fig. 2:**
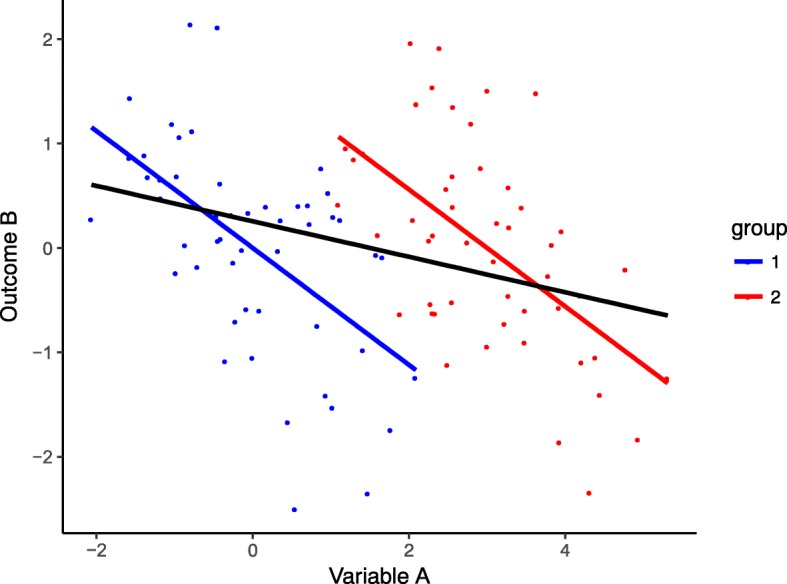
Demonstration of correlation bias by combining dissimilar groups. In randomly sampled groups showing the same strong inverse correlation (blue and red regression line) but slightly shifted centres, a combined analysis (black regression line) - unlike the correct analysis by individual groups - weakens the correlation. Distinguishing between averaged and typical outcomes is particularly important for parameters with known high individuality such as thyroid hormones

Using the observed historical narrower therapeutic range for an individual patient we note that the treatment targets may overlap for patients in a group. If that is true the general assumption that maintaining TSH anywhere within its broad reference limits to routinely achieve a satisfactory outcome for each and every patient may be ill advised. We have refuted the applicability of treatment targets based on the consideration of the reference ranges in the healthy population, by demonstrating dissociations between FT3 and FT4, and FT3 and TSH in LT4-treated athyreotic patients, and documenting altered equilibria between the hormones on LT4, compared to the healthy state [27, 56]. Others have arrived at similar conclusions [57]. In laboratory diagnostics, the high individuality of TSH and thyroid hormones has long been recognised since the pioneering work of Andersen and colleagues [58]. However, this applies equally to the statistical analysis of associations involving thyroid parameters. Data clustering, be it in groups with similar properties or in subjects where multiple measurements are obtained over time, potentially masks the true relationship, abolishing the strong associations at the group level when the data are combined for analysis. This phenomenon, known as Simpson’s paradox, is readily demonstrated with a fictitious random sample of two groups with a slightly shifted centre showing the same strong inverse correlation. Unlike the correct analysis by individual groups, a combined analysis of the total cohort artificially weakens the correlation (Fig. 2). The analytical distinction between the averaged versus the typical outcome is clinically relevant for all thyroid drug trials, independently of evidence class and study design, because doctors are naturally more interested in the latter.

In a large retrospective longitudinal study, relying on a multilevel model and accounting for both within-subject and between-subject variation, symptomatic outcomes were associated with serum FT3 concentrations, and differed according to the placement of biochemical parameters within the reference range or noticeably beyond its limits in the case of TSH and FT4 [50]. Treatment-related displacement of the equilibria between thyroid parameters, wide variations in the biochemical treatment response, and individually adjusted dose requirement pose particular challenges for thyroid trials [27, 45, 59, 60]. Demonstration of averaged equivalency cannot therefore be a satisfactory analytical goal [61].

Accordingly, the value of statistical evidence derived from historical meta-analyses [62–66] and RCTs on the acceptability of T3/T4 combination therapy is severely weakened and requires careful reconsideration [60]. Many RCTs were conducted with inferior quality of life instruments available at the time and relied on statistical techniques both less suited for highly individual parameters and in addition susceptible to Simpson’s paradox. Using the overall preference expressed by patients at the end of double-blind studies as a proxy, patients mostly favoured T3/T4 combination therapy [52]. A thyroid-specific QoL has only recently been developed and validated [51]. Simpson’s paradox (also known as amalgamation bias or collider stratification bias) may explain, at least in part, why otherwise well-performed studies failed to provide a convincing relationship between symptoms and thyroid function tests [27, 54, 59–61]. The paradox is a relevant factor for the relationship of patient complaints, biochemical markers and treatment response to LT4 [60]. This bias - unless properly accounted for statistically - dissociates the personal treatment responses from the statistical group effect, thereby masking individual treatment success or failure in an unchanged grouped outcome. A lack of group to individual generalizability has been increasingly recognized in other fields, requiring explicit testing for equivalence of processes both at the individual and group level [61].

Trials purporting to relate TSH and thyroid hormone levels to the incidence of osteoporosis and atrial fibrillation fall under the same fundamental caveats [24, 27]. In particular, the Rotterdam study has shown that within the euthyroid range the prognostic implications of thyroid hormones and TSH differ, and, that TSH measurements therefore cannot substitute for FT4 concentrations in predicting the risk of atrial fibrillation [67]. The cause of atrial fibrillation poses a complex problem, as its occurrence has been physiologically and statistically associated with both high and low FT3 concentrations [68]. Thyrotoxicosis due to exogenous thyroid hormone intake and endogenous hyperthyroidism have different physiological roots. This traditional distinction should be noted because the interrelationships between TSH and thyroid hormones differ on LT4 treatment from those in thyroid health [24, 56, 57, 59, 69]. This may explain why a prospective study measuring surrogate markers of thyroid tissue effects in athyreotic patients found a slightly suppressed TSH to be optimum for these patients rather than constituting overtreatment [57]. This problem is paralleled in FT4 measurements, which also overlap significantly at the hypothyroid-euthyroid borderline, both in untreated states and even more so in LT4-treated patients [24, 26, 59, 67]. However, this neither implies that TSH suppression is universally desirable, nor that a suppressed TSH is without risk [24]. Rather TSH by itself, unaccompanied by measurements of FT4 and FT3, is an unsuitable risk measure in LT4-treated patients, displaying considerable inherent uncertainty in an individual about the risk - benefit ratio for TSH values close to the lower reference limit [27, 69]. Taken together, a combination of nonspecific complaints, statistical group-to-individual bias and limited diagnostic performance of TSH testing obfuscates the transition between diseased and healthy state and fosters disagreement of interpretation depending on the respective focal points.

Serious correction of scientific evidence is not unprecedented in medicine. Notably, some cholesterol trials have undergone re-interpretation, reversing previous conclusions, following re-analysis of recovered crude data with improved statistical methods [70]. Market retraction of the antidiabetic drug rosiglitazone is just one noteworthy example of an initially overlooked effect reversion due to Simpson’s paradox [71]. New studies could be performed in the light of changes in the treatment habits, consequential shifts in symptom reporting and the complaint spectrum as well as recent developments in statistical analysis which favour greater stratification of disease aetiology and individual outcome before commencing suitable analytic procedures. Emphasis should be more strongly concentrated on personalised treatment strategies, reflected by appropriate protocols and statistical instruments favouring multilevel analysis or latent class hierarchical models. Range-based use of biochemical thyroid parameters, though having an essential role in diagnosis, should not automatically dominate patient presentation and surrogate markers for tissue T3 effects [26, 57, 60, 72, 73].

When rejecting patient preference as an objective criterion, standard LT4 and combination therapy performed equally on average on QoL measures in several metanalyses [62–66]. However, heterogeneity of the observed treatment response and collider stratification bias require targeting homogenous subgroups and performing statistical latent class analysis [60, 61]. This may identify patients that preferentially benefit from the two modalities [60]. TSH and FT3 dissociate under LT4 treatment, particularly in athyreotic patients where equilibria are formed between TSH and FT4/FT3 different from the healthy state [56, 57, 59]. Poor T3 converters with persisting symptoms may thus be the most suitable candidates for trials of T3/T4 combinations. T3 addition may also avoid LT4 dose escalation resulting in T4 excess, as T4 has been implicated in non-genomic actions, not mediated via T3, such as actin-related cell migration [74].

Following the timeless wise words of Paracelsus “Dosis solum facit venenum” (“Only the dose makes the poison”). and in keeping with the historic practice to adjust LT4 dose based on a metabolic marker, individual dosing regimens and personalised treatment targets have to be reconsidered [27]. This is another area where current TSH based LT4 dosing guidelines fall short, as carefully conducted experiments in rodents, which cannot be performed in humans, have shown [72, 73]. LT4 monotherapy was unable to restore euthyroidism at the level of various tissues in the animals despite bringing TSH within its reference range [72, 73].

## Conclusions

Until the situation is clarified all currently available treatment options should remain on the table and the focus should remain on facilitating the free choice of prescriptible treatment options rather than imposing new restrictions. The biochemically based reason for the rise in patient complaints has to be addressed, not a shift on to them of blame and burden of proof.

This invites a resume of the current state of affairs.

It appears that what we are witnessing constitutes an unprecedented historic change in the diagnosis and treatment of thyroid disease, driven by over-reliance on a single laboratory parameter TSH and supported by persuasive guidelines. This has resulted in a mass experiment in disease definition and a massive swing of the pendulum from a fear of drug-induced thyrotoxicosis to the new actuality of unresolved designation of hypothyroidism. All of this has occurred in a relatively short period of time without any epidemiological monitoring of the situation. Evidence has become ephemeral and many recommendations lag behind the changing demographic patterns addressing issues that are no longer of high priority as the pendulum has already moved in the opposite direction. In a rapidly changing medical environment, guidelines have emerged as a novel though often over-promoted driver of unprecedented influence and change. Treatment choices no longer rest primarily on the personal interaction between patient and doctor but have become a mass commodity, based on the increasing use of guidelines not as advisory but obligatory for result interpretation and subsequent treatment. Contrary to all proclaimed efforts towards a more personalised medicine, this has become a regulated consumer mass market as with many other situations. This is of little benefit to patients who will continue to complain, and with some justification, that the medical profession is not listening, thereby abandoning one of its primary functions in the doctor-patient relationship.

## Abbreviations

FT3: free triiodothyronine
FT4: free thyroxine
HPLC: high pressure liquid chromatography
LT4: levothyroxine
QoL: quality of life
RCT: randomised clinical trial
T3: total triodothyronine
T4: total thyroxine
TSH: thyroid stimulating hormone
